# Homozygosity mapping identified loci and candidate genes responsible for freezing tolerance in *Camelina sativa*

**DOI:** 10.1002/tpg2.20318

**Published:** 2023-03-09

**Authors:** TM Shaikh, Mukhlesur Rahman, Timothy Smith, James V. Anderson, Wun S. Chao, David P. Horvath

**Affiliations:** ^1^ Department of Plant Sciences North Dakota State University Fargo ND USA; ^2^ USDA/ARS Genetics and Animal Breeding Clay Center NE USA; ^3^ USDA/ARS, Sunflower and Plant Biology Research Unit, Edward T Schafer Agricultural Research Center Fargo ND USA

## Abstract

Homozygosity mapping is an effective tool for detecting genomic regions responsible for a given trait when the phenotype is controlled by a limited number of dominant or co‐dominant loci. Freezing tolerance is a major attribute in agricultural crops such as camelina. Previous studies indicated that freezing tolerance differences between a tolerant (Joelle) and susceptible (CO46) variety of camelina were controlled by a small number of dominant or co‐dominant genes. We performed whole genome homozygosity mapping to identify markers and candidate genes responsible for freezing tolerance difference between these two genotypes. A total of 28 F3 RILs were sequenced to ∼30× coverage, and parental lines were sequenced to >30–40× coverage with Pacific Biosciences high fidelity technology and 60× coverage using Illumina whole genome sequencing. Overall, about 126k homozygous single nucleotide polymorphism markers were identified that differentiate both parents. Moreover, 617 markers were also homozygous in F3 families fixed for freezing tolerance/susceptibility. All these markers mapped to two contigs forming a contiguous stretch of chromosome 11. The homozygosity mapping detected 9 homozygous blocks among the selected markers and 22 candidate genes with strong similarity to regions in or near the homozygous blocks. Two such genes were differentially expressed during cold acclimation in camelina. The largest block contained a cold‐regulated *plant thionin* and a putative *rotamase cyclophilin 2* gene previously associated with freezing resistance in arabidopsis (*Arabidopsis thaliana*). The second largest block contains several *cysteine‐rich RLK* genes and a cold‐regulated *receptor serine/threonine kinase* gene. We hypothesize that one or more of these genes may be primarily responsible for freezing tolerance differences in camelina varieties.

## Core Ideas

- We refined a homozygosity mapping procedure for freezing tolerance loci in camelina.
- We identified several regions on chromosome 11 of camelina associated with freezing tolerance.
- Candidate genes include cysteine‐rich receptor‐like protein kinases and receptor serine/threonine kinases.

**Abbreviations**
LED: light‐emitting diode
PacBio HiFi: Pacific Biosciences high fidelity
QTL: quantitative trait loci
RIL: recombinant inbred line
RLK: receptor‐like protein kinase
SNP: single nucleotide polymorphism

## 1 INTRODUCTION

Camelina (*Camelina sativa*), also referred to as false flax, gold of pleasure, or linseed dodder is an under‐used oilseed crop from the Brassicaceae family (Berti et al., 2016; Vollmann et al., 2007; Kagale et al., 2014). Camelina is an emerging biofuel crop (Moore et al., 2017) that has both winter and spring biotypes (Anderson et al., 2018). In more moderate regions, it can be planted as a winter annual, but the spring variety, which does not require vernalization, is generally preferred by growers in the Northern Great Plains because winter survival is inconsistent for most available commercial varieties (Gesch & Archer, 2013; Berti et al., 2016). Camelina has reportedly been cultivated in Europe for over 5000 years (Bouby, 1998) but has gained notoriety recently as a potential source of omega‐3 fatty acids and for producing niche oil and animal feed stocks (Malik et al., 2018; McVay & Lamb, 2008; Luo et al., 2020). The major use of camelina oil is for a hydroprocessed renewable jet fuel (Berti et al., 2016; Moore et al., 2017). The US military revealed that camelina oil met all jet engine performance standards and reduced greenhouse gas emissions significantly (Shonnard et al., 2010). Like other winter Brassica oilseed crops, camelina sets seed early in the growing season and produces good yields (743–2300 kg ha^−^^1^ in west central Minnesota) under harsh dry and cold environments with low inputs—even in saline soil (Gesch, 2014). Combined, these attributes make it an excellent choice for cropping in the Northern Great Plains region (Gesch & Cermak, 2011; McVay & Lamb, 2008). In addition, camelina shows potential as a weed suppressing cover crop (Saucke & Ackermann, 2006) that reduces soil nutrient losses (Berti et al., 2016) and offers an early season nectar source for pollinators (Eberle et al., 2015). Camelina also has the advantage of a short growth cycle (85−100 days) crop (Abdullah et al., 2016; McVay & Lamb, 2008) and thus, has promise for dual cropping opportunities for North Dakota (ND) growers (Berti et al., 2016; Gesch & Archer, 2013). Despite having potential, relative to canola, there has been little research to improve biotic and abiotic stress tolerance (Berti et al., 2016).

Camelina is allohexaploid (2n = 40) in nature and shows close syntenic relation to arabidopsis (*Arabidopsis thaliana*). A draft reference sequence of the camelina genome was first released in 2014 and it appears to have an ∼782 Mb genome (Kagale et al., 2014). Anderson et al. (2018) also released whole genome and transcriptome sequence data from a winter biotype “Joelle’” and summer biotype “CO46” at ∼25× coverage. The camelina chromosome scale reference genome reported a total of 89,418 annotated protein‐coding genes (Kagale et al., 2014) and a transcriptome atlas with a coverage of 88% of the annotated genes (Kagale et al., 2016). This available reference genome, together with camelina's ability to be transformed using agrobacterium with a floral dip procedure (Liu et al., 2012; Lu & Kang, 2008) has made camelina a unique crop species for genomic studies and has opened the opportunity to introduce useful agronomic traits through genetic engineering (Abdullah et al., 2016; Anderson et al., 2019; Berti et al., 2016). The camelina genome is hypothesized to be composed of an allotetraploid sub‐genome with seven chromosomes each and a diploid sub‐genome with six chromosomes (Kagale et al., 2014). Although having reasonable synteny with arabidopsis, the presence of three copies of homologous genes (allohexaploid nature), highly undifferentiated polyploidy, and small fractionation bias within its genome has made camelina more complicated for studies compared to arabidopsis (Anderson et al., 2018; Horvath et al., 2019; Kagale et al., 2014). Camelina poses a low level of genetic diversity, and a majority of the domesticated cultivars are spring type, whereas most of its wild relatives are winter type (Berti et al., 2016; Chaudhary et al., 2020).

**Core Ideas**

- We refined a homozygosity mapping procedure for freezing tolerance loci in camelina.
- We identified several regions on chromosome 11 of camelina associated with freezing tolerance.
- Candidate genes include cysteine‐rich receptor‐like protein kinases and receptor serine/threonine kinases.

Freezing stress significantly impairs plant growth and productivity and is often fatal in extreme conditions (Thomashow, 1990, 1999). Freezing damage is initiated by extracellular ice crystal formation, which later causes irreversible damage to plants through extreme cellular dehydration and cellular dysfunction (Ding et al., 2019; Fiebelkorn et al., 2018; Pearce, 2001; Thomashow, 1990). The ability of plants to survive freezing is advantageous for winter cover crops (Wilke & Snapp, 2008) and unraveling the freezing mechanisms both at genetic and physiological level is a well‐researched phenomenon (Soorni et al., 2017). Plants can reduce freezing damage if first subjected to a period of time at low but non‐freezing temperatures (Thomashow, 1990) often referred to as cold acclimation. Camelina can survive exceptionally low temperatures (−23°C for 8 h) following acclimation (Berti et al., 2016). Cold acclimation results in increased levels of protective proteins such as late embryogenesis abundant (LEA) proteins, anti‐freeze proteins (AFPs), cold shock proteins (CSPs), and synthesizing hydrophilic compounds such as proline and soluble sugars (Ding et al., 2019; Guy et al., 1994; Thomashow, 1990). In arabidopsis, cold acclimation also induces a family of transcription factors known as C‐REPEAT BINDING FACTORs (CBFs), which in turn, regulates a set of COLD‐REGULATED (COR) genes (Thomashow, 1990). This signaling process is often referred to as the CBF regulon (Ding et al., 2019; Jaglo‐Ottosen et al., 1998). However, a transcriptomic study on two contrasting camelina varieties Joelle (a freezing tolerant winter biotype) and CO46 (a freezing sensitive spring biotype) revealed that the activation of CBF regulon was significantly induced in both varieties after 8 weeks of cold acclimation, although there were differences in the level of induction (Anderson et al., 2022; Horvath et al., 2019; Wang et al., 2021). These studies also indicated several sets of transcription factors and other potential regulatory factors besides CBFs were associated with differential survival following freezing in camelina. Elucidating the freezing survival mechanisms in camelina may lead to increased agricultural production of camelina and potentially enhance winter hardiness for other significant brassica species such as canola through genetic engineering approaches with camelina genes (Berti et al., 2016; Soorni et al., 2017).

**FIGURE 1:**
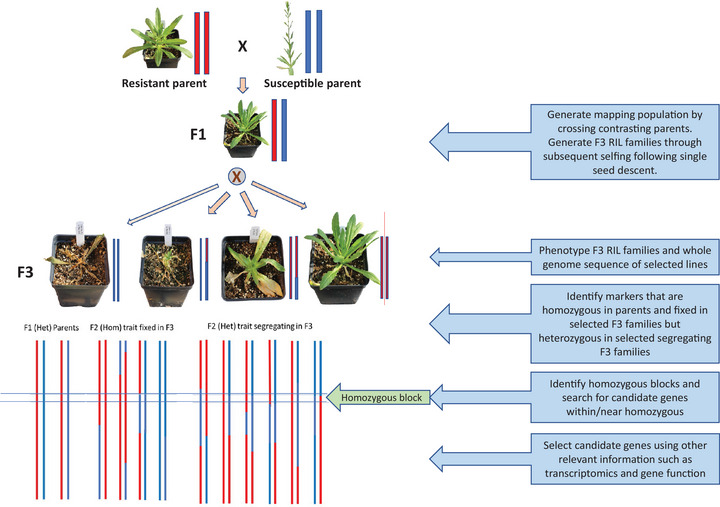
Schematic workflow for homozygosity mapping, homozygous block identification, and candidate gene identification for freezing tolerance. The red lines represent the chromosome of parent that was homozygous for the trait of interest (freezing tolerant) and the blue lines represent the chromosome of the other parent which was homozygous for the wild type trait (freezing sensitive). Chromosomes with red and blue segments represent crossovers that occurred in the generation of the F2 individuals. The gene(s) of interest must be within the region that was both homozygous in the families of the F3 recombinant inbred lines (RIL) which were fixed for that trait of interest, and which were heterozygous in the F3 RIL families that were segregating for the trait of interest (the region depicted between the two horizontal lines).

Homozygosity mapping, also known as autozygosity mapping, is considered an efficient gene mapping procedure to identify recessive disorders/traits in inbreed families (Gholipoorfeshkecheh et al., 2020; Seelow et al., 2012). The principal mechanism behind the homozygosity mapping is to trace candidate genes by identifying homozygous regions in the affected individuals and identify causal mutation for a disorder through genome wide screening using a high‐density single nucleotide polymorphism (SNP) based platform (Gholipoorfeshkecheh et al., 2020). A schematic for how this works is shown in Figure 1. Although homozygosity mapping is similar to bulk segregant analysis (Li et al., 2021; Michelmore et al., 1991; Wang et al., 2021), it differs in that rather than just selecting two extremes of the population with no regard to genotype, only individuals known to be homozygous for the traits are selected and compared to individuals known to be heterozygous. Also, rather than just looking for statistical associations between markers and the trait of interest, the goal of homozygosity mapping is to identify contiguous stretches of DNA that are homozygous in all individuals that are fixed for the trait, and which are heterozygous in all individuals where the trait is segregating. Homozygosity mapping has been successfully applied in humans and livestock research, but there are limited applications of this technique in plant species. Studies on several human recessive diseases had revealed that the chances of identifying the candidate genes within a homozygous block is over 90% (Soorni et al., 2017). Since homozygosity mapping is quite dependent on the availability of a reference genome, current cost‐effective genotyping technology has opened the opportunity to explore this technique in several plant species (Kumar et al., 2020).

The apparently limited genetic loci controlling the major differences in freezing tolerance between Joelle and CO46, and the availability of a reference genome for this species makes it an ideal target to attempt homozygosity mapping to identify the primary causal genes underlying the freezing tolerance differences. In this study, we attempted to identify a reliable set of molecular markers through whole genome sequencing of winter biotype Joelle and summer biotype CO46 and the limited number of derived F3 RIL families that appeared to be homozygous for the loci controlling freezing tolerance or susceptibility, respectively. Our objective was to identify homozygous blocks and to look for causal candidate genes within and near these loci. Resulting candidate gene(s) should indicate potential targets to improve freezing tolerance in camelina and its related species such as canola.

## 2 MATERIALS AND METHODS

### 2.1 Plant materials and phenotyping of F3 RIL

A reciprocal cross between two parental cultivars with contrasting freezing tolerance: Joelle (freezing tolerant) and CO46 (freezing susceptible), generated and 255 F2 seeds. Thus, F3 RIL families were generated by selfing the F2 seeds. Seeds from all 255 F3 segregating RILs were grown in 500 mL pots containing Sun‐shine mix #1 (Fisons Horticulture Inc., Bellevue, WA, USA) under greenhouse conditions (16 h photoperiod with supplemental halogen lighting at 22–25˚C) with daily watering and weekly fertilizer application to the 6–8 leaf stage. Additionally, 254 F7 RIL families derived by single seed decent from these F2 seeds were also similarly tested to confirm the homozygosity of the phenotypes from the fully tolerant and susceptible F3 RIL families (one line was lost during generation of the F7 RIL populations).

**FIGURE 2:**
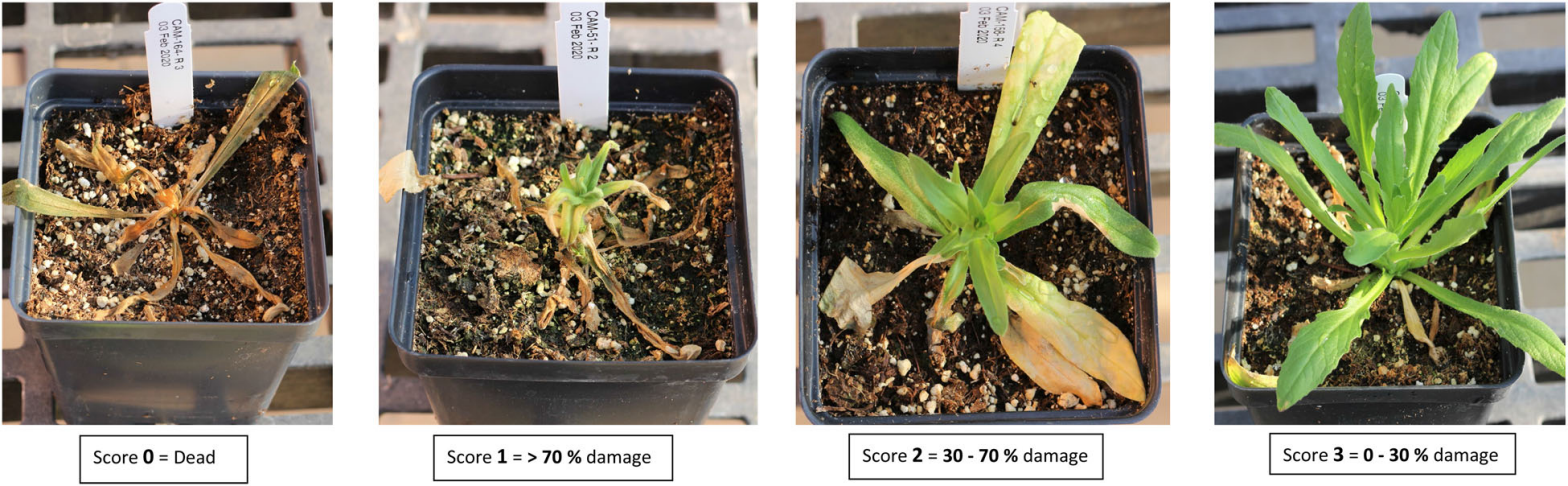
Photos of freezing damage with scale used in phenotyping.

Plants were then cold acclimated for 6 weeks in temperature‐controlled walk‐in growth chambers at 5˚C under 12 h photoperiods augmented with full spectrum LED lighting with an average intensity of 4500 lux at plant level prior to freezing. Acclimated plants were then subjected to a freezing treatment consisting of a linear ramp up to 15˚C at noon followed by a linear ramp down in temperature to 5˚C at 6:00 p.m. with lights on. Lights were turned off and plants were ramped down to −15˚C at 4:00 a.m. and held at that temperature for 4 h. Lights were then turned on, and plants were rapidly ramped up to 0˚C over the next hour and then back to 15˚C by noon. Such conditions are designed to mimic a hard early morning frost in the fall or early spring. Plants were then transferred to the greenhouse and scored for survival after 2 weeks using a visual survival scale of 0–3 (0 = dead, 1 = more than 70% leaf damage but surviving meristematic tissue, 2 = 30%–70% leaf damage and surviving meristematic tissue, 3 = 0%–30% leaf damage and surviving meristematic tissue) (Figure 2). Based on the above‐mentioned phenotypic scale, freezing tolerant families that were fixed for freezing tolerance/susceptibility and families that appeared to still be segregating for freezing tolerance/susceptibility were identified.

### 2.2 Experimental design

A complete randomized design (CRD) was used for each experimental run with 12 technical replicates for each of the 255 F3 families, and 6 technical replicates for the two parental lines as positive and negative controls. After completing one round of phenotyping, we selected all the promising lines that were seemingly tolerant, susceptible, and segregating. We performed phenotyping one additional time for those selected lines. Based on the phenotyping data of the F3 RIL population, we selected 6 families with high ratios of freezing tolerance, 6 families with low ratios of freezing tolerance, and 16 families that appeared to be segregating for freezing tolerance for whole genome sequencing in the F3 families.

### 2.3 DNA extraction and library preparation

To perform whole genome sequencing, young leaf tissues from 12 individual plants of each of the 28 selected families, along with leaf tissue from 6 plants of each parental variety, were collected and separately pooled. All samples were frozen immediately in liquid nitrogen and kept at −80^°^C prior to DNA extraction. Pooled samples were homogenized, and DNA was extracted from each pool using a standard CTAB extraction (Doyle & Doyle, 1990). DNA concentrations were quantified using a NanoDrop 2000 (Thermo Fisher Scientific, USA) and the DNA integrity was evaluated by electrophoresis on 1% agarose gels. The high‐quality DNA was used to produce genomic libraries using the NEBNext Ultra II FS DNA Library prep Kit for sheared DNA (200–450 bp) Illumina sequencing (catalogue #E7805L) according to manufacturer's protocols with the following minor modifications. Ampure beads were used for initial size selection followed by size selection using Pippin Prep gels (CDF1510), 2% Dye‐Free, range 100–600 bp, and the peak between 200 and 300 bp was selected. Additionally, Pacific Biosciences high fidelity (PacBio HiFi) was used to sequence the parental lines (∼40× coverage for CO46 and ∼28× coverage for Joelle). The resulting sequences from both parents were assembled independently using the program Hifiasm with default parameters. The resulting ∼5000 contigs from CO46 along with an additional ∼1000 contigs from Joelle that did not appear to have any matches to the CO46 contigs were combined to serve as a new reference genome set (supplemental file S1) for mapping of short read sequences. The program MUMmer4 (Marçais et al., 2018) was used with default parameters to align CO46 contigs to the published camelina reference genome and to identify those contigs unique to Joelle. Additionally, BlastN was used to map previously published camelina gene sequences to this new reference genome set.

### 2.4 Whole genome sequence read mapping and SNP calling

Tagged genomic cDNA libraries were pooled and sent to Novogene Genomic Service & Solutions, China for whole genome sequencing at ∼30× coverage with 150 base paired end reads using Illumina NovaSeq technology. Raw whole genome sequenced FastQC data was quality trimmed and contaminating primers were removed using BBMAP (Bushnell, 2014) sub‐programs (supplemental file S2). Briefly, trimming was done using BBDuk maintaining a parameter of minlen = 70, qtrim = rl, trimq = 20, ktrim = r, k = 28, mink = 12, hdist = 1, ref = /bbmap/resources/adapters.fa. Trimmed and filtered sequenced reads were then aligned to the assembled camelina contigs (supplemental file S1). In addition to these 30 libraries, sequence data from a previous whole genome sequencing project that provided additional files with greater than 25× coverage of both parental lines (Anderson et al., 2018) were added to the downstream analyses. Mapping of fragments was done using Bowtie2 with default parameters (supplemental file S3). This reference‐guided approach was used to generate a vcf file using BCFtools software. All the procedures were completed using python bash scripts on SSH Linux platform. The resulting vcf file was then filtered for calling high‐quality SNPs using VCFtools software, where read counts smaller than 40 for either major or minor allele were discarded and max‐missing = 0.5, maf = 0.05, and minimum DP of 10 were maintained. The raw reads have been deposited in the SRA database (accession number PRJNA880815).

### 2.5 Identifying markers and candidate genes

From the above‐mentioned quality filtered SNP vcf file, we identified markers that were homozygous but divergent in at least three of the four parental lines in both the current sequencing run and those from a previous whole genome sequencing of the two parental lines (Anderson et al., 2018). This resulted in 125,899 markers from ∼2 million seemingly high‐quality raw SNP markers from the 28 F3 families and both sets of sequence from parental lines. These markers were then extracted from the vcf files of the F3 RIL families. Markers that were homozygous in both independent sequencing runs for the parental lines, and which were homozygous in the corresponding tolerant/susceptible F3 families, and which were also heterozygous in the F3 families segregating for freezing tolerance were identified by a simple sorting algorithm in excel. Briefly, from our identified set of markers, we screened for homozygous blocks using block length with a threshold of 50 kb distance apart. We defined a sliding window size of 50 kb distance and quantified the number of markers within each window. By dividing the number of markers per window by the total number of markers in our study, we obtained the relative frequency of markers per window value. Those relative frequency of markers per window values were plotted to visualize peaks indicating homozygous block. We did not consider a peak as homozygous block if the peak forming windows had less than 10 markers or a relative frequency of markers designated as homozygosity score of less than 0.016. Genes present within or adjacent to the homozygous blocks were identified using the BlastN‐created map described above. We also examined our homozygous blocks with the syntenic regions of other brassica species for freezing tolerance/susceptibility by mapping sequences within our blocks to loci in other species by sequence similarities and gene order and location from various sources and genome browsers. The workflow of homozygosity mapping is depicted in Figure 1.

## 3 RESULTS

### 3.1 Phenotyping analysis to select F3 RILs for whole genome sequencing

**FIGURE 3:**
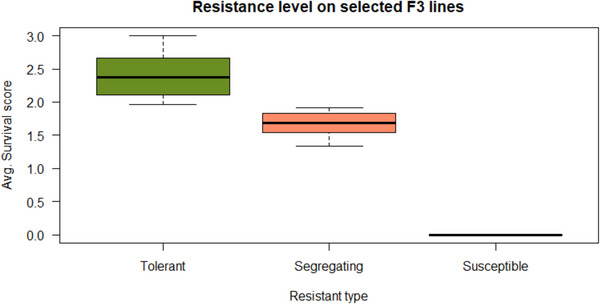
Boxplot showing phenotypic distribution and resistance level of selected 28 F3 RIL families and their parents (based on average survival score from two experiments) that were advanced for whole genome sequencing.

Phenotyping all F3 RIL families for freezing tolerance indicated that there was considerable variation in the response to freezing conditions between experiments. Thus, multiple experimental runs were needed to identify F3 families that consistently showed limited segregation for freezing tolerant/susceptible phenotypes. This analysis eventually identified three non‐segregating F3 families with freezing tolerance similar to the Joelle parent, and 54 families with weak freezing tolerance similar to the CO46 parent. There were 198 families that appeared to still be segregating for freezing tolerance (Table 1). We selected 28 F3 families for whole genome sequencing based on survival score for freezing tolerance. Among those selected F3 families, three had >90% freezing tolerant individuals and three had >75% freezing tolerant individuals in both experimental runs, six appeared to have a high percentage of freezing sensitive individuals (100% in both experimental runs), and the remainder appeared to be segregating for tolerance/susceptibility (Figure 3). A list of selected accessions is shown in supplemental file S4. However, analysis of the F7 RIL mapping population indicated that only two of the six selected lines were truly homozygous for freezing tolerance and only two maintained homozygosity for freezing susceptibility (data not shown), suggesting that only a subset of the 12 selected F3 families were likely fixed for freezing tolerance/susceptibility.

**TABLE 1:** Phenotyping distribution of all 254 F3 RILs

Type of tolerance	Segregation ratio	Number of RIL families
Highly tolerant	1:0	3
Moderately tolerance	∼3:1	43
Intermediate tolerance	∼1:1	62
Moderately susceptible	∼1:3	93
Highly susceptible	0:1	54

*Note*: Ratios shown are the number of freezing tolerant individuals (with survival scores greater than 2) to freezing susceptible individuals (survival scores less than 2) from each F3 RIL family.

### 3.2 Analysis of sequenced data and discovery of homozygous markers

**FIGURE 4:**
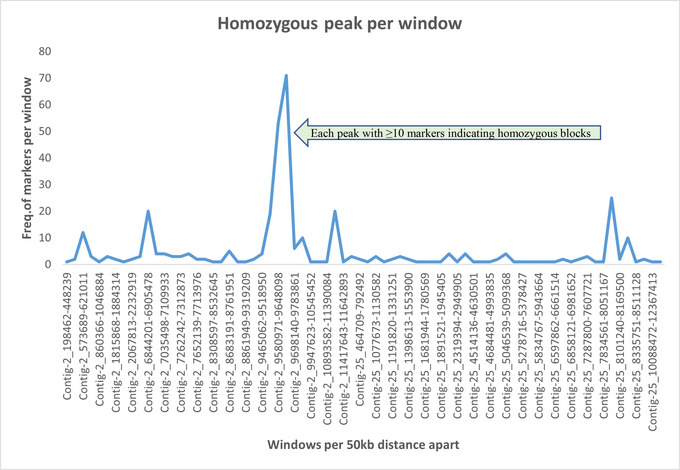
Peak indicating homozygous block based of relative frequency of markers per window of 50 kb distance apart (generated from 617 markers).

We obtained ∼2 million seemingly high‐quality SNP markers from the 28 F3 families and both sets of sequence from parental lines. Among them 125,899 markers were homozygous in both parents and consistently differentiated the two parents in the earlier study by Anderson et al. (2018). Two families did not have sufficient sequencing depth and were dropped from the analysis. Additionally, several families were dropped because their phenotype was divergent from what was expected in the F7 generation or were lost during advancement to the F7 generation. This left five freezing tolerant lines (three were fully tolerant with a survival score of 3 and the other two were highly freezing tolerant, showing maximum survival score of close to 3), four freezing susceptible lines, and six lines that were likely still segregating for freezing tolerance. Additional filtering for high‐quality SNPs using VCFtools software resulted in 57,251k SNP markers which were homozygous in both independent sequencing runs of the two parents (the current run and the previous run described by Anderson et al., 2018). Of these, only 617 markers had limited or no missing data and were homozygous in all families fixed for freezing tolerance/resistance and heterozygous in the families that were segregating for freezing tolerance. Intriguingly, these 617 markers mapped to just two contigs (contig 2 and contig 25) (supplemental file S5). Out of the 617 markers, contig 2 and contig 25 had 365 and 252 markers, respectively. Unexpectedly, many of the other markers on these contigs, although homozygous in the parents, still showed segregation in the four non‐segregating progeny. Thus, we looked for blocks of homozygosity where closely linked markers (within a block length of 50,000 bases) were found in consecutive stretches of 10 or more markers (see yellow highlighted regions in supplemental file S5). However, a total of seven and two closely linked homozygous blocks were detected on contigs 2 and 25, respectively. These resolved into four distinct peaks on contig 2 and one peak on contig 25 (Figure 4). It should be noted that both contigs map contiguously on Chromosome 11 in the previously published camelina reference genome (Table 2).

**TABLE 2:** Camelina genes that map to regions of contigs 2 and 25 which contain extended stretches of markers that are homozygous in both parent and offspring that are fixed for the trait, and which were heterozygous in offspring that appeared to still be segregating for the trait

ID	CGI	Contig	Start position	End position	Distance	Distance	AGI	Function
Scontig‐2 611735	Csa12g061530	contig 2	616724	616336	4989	4601	AT4G13920	Receptor‐like protein 50
Scontig‐2 6844201	Csa11g045230	contig 2	6841258	6840861	2943	3340	AT5G35930	AMP‐dependent synthetase and ligase family protein
Scontig‐2 6865515	Csa11g045250	contig 2	6862237	6861901	3278	3614	AT5G35960	Protein kinase family protein
Scontig‐2 6888981	Csa11g045260	contig 2	6887660	6887515	1321	1466	NA	NA
Scontig‐2 6890761	Csa11g045270	contig 2	6891014	6891310	253	549	NA	NA
Scontig‐2 9532659	Csa11g052070	contig 2	9532839	9532415	180	244	AT5G38260	Protein kinase superfamily protein
Scontig‐2 9538834	Csa11g052080	contig 2	9539171	9538441	337	393	AT5G38280	PR5‐like receptor kinase
Scontig‐2 9648098	Csa11g052140	contig 2	9643299	9643565	4799	4533	AT3G56070	Rotamase cyclophilin 2
Scontig‐2 9648098	Csa11g052150	contig 2	9646410	9645838	1688	2260	AT5G38310	Unknown protein
Scontig‐2_9690452	Csa11g052160	contig 2	9692525	9692777	2073	2325	AT5G38317	Low‐molecular‐weight cysteine‐rich 58
Scontig‐2 9783861	Csa11g052230	contig 2	9782694	9782921	1167	940	AT1G66100	*Plant thionin*
Scontig‐2 11390084	Csa11g055590	contig 2	11388927	11388723	1157	1361	AT5G39520	Protein of unknown function (DUF1997)
Scontig‐25 792492	Csa00506s030	contig 25	788032	788391	4460	4101	AT4G15053	Protein of unknown function (DUF239)
Scontig‐25 792492	Csa00506s040	contig 25	790563	790226	1929	2266	AT1G74690	IQ‐domain 31
Scontig‐25 8082177	Csa11g022790	contig 25	8080989	8080272	1188	1905	AT4G23190	Cysteine‐rich RLK (RECEPTOR‐like protein kinase) 11
Scontig‐25 8082177	Csa12g033370	contig 25	8082148	8081897	29	280	AT4G23210	Cysteine‐rich RLK (RECEPTOR‐like protein kinase) 13
Scontig‐25 8082177	**Csa11g022780**	contig 25	8082797	8082555	620	378	AT4G23210	Cysteine‐rich RLK (RECEPTOR‐like protein kinase) 13
Scontig‐25 8098455	Csa12g033360	contig 25	8095101	8094400	3354	4055	AT4G18250	Receptor serine/threonine kinase, putative
Scontig‐25 8098455	**Csa11g022770**	contig 25	8095101	8094404	3354	4051	AT4G18250	Receptor serine/threonine kinase, putative
Scontig‐25 8098455	Csa12g033320	contig 25	8095101	8094931	3354	3524	AT4G18250	Receptor serine/threonine kinase, putative
Scontig‐25 8101208	Csa11g022760	contig 25	8104809	8103861	3601	2653	AT4G23220	Cysteine‐rich RLK (RECEPTOR‐like protein kinase) 14
Scontig‐25 8251725	Csa11g022560	contig 25	8253944	8253561	2219	1836	AT4G23380	Protein of unknown function (DUF239)

*Note*: Bolded genes indicate those that were shown to be differentially expressed between the freezing tolerant and freezing susceptible genotypes following cold acclimations in a previous study (Anderson et al., 2022).

### 3.3 Discovery of probable candidate genes for freezing tolerance or susceptibility

We identified 22 camelina genes that had homology to sequences within or very near (within 5 kb upstream or downstream) of our selected homozygous blocks (Table 2). Additionally, we identified two genes within or near our homozygous blocks that were differentially expressed (Table 2—green highlighted) between Joelle and CO46 after cold acclimation in previous studies (Anderson et al., 2022). The largest peak was on contig 2 between the position of 9,518,950 and 9,698,140. Three contiguous blocks of 50 kb distances fall within this peak. Surprisingly, of the four peaks observed on contig 2, most were not enriched with genes previously reported to be involved in freezing tolerance. However, on contig 2, one block within the large peak contained four genes located between position 6,844,201 and 6,905,478 with two genes of interest. One of these genes is similar to a *rotamase cyclophilin 2* that has been implicated in freezing tolerance in arabidopsis (Weng et al., 2020), and the other encodes a *plant thionin* gene that was differentially expressed following cold acclimation in camelina (Anderson et al., 2022). The peak on contig 25 contained two blocks with six genes (Table 2). Fortuitously, these six genes form two clusters of three *cysteine‐rich RLK* (RECEPTOR‐like protein kinase) genes and three cold‐regulated *receptor serine/threonine kinase* gene each. However, we cannot rule out that other loci within or outside of these two contigs might also be playing a role in the differences in freezing tolerance observed in the parental lines.

## 4 DISCUSSION

Extreme winters and short summer growing seasons are a common phenomenon in Northern Great Plain regions where a relatively short growing season and good winter hardiness of winter camelina have made it an excellent choice for growing as a cover crop (Berti et al., 2017a; Gesch & Archer, 2013; Gesch & Cermak, 2011; Gesch et al., 2014, 2011). However, in the Northern Great Plains, summer biotypes of camelina are predominantly grown because winter kill is still occasionally problematic (Berti et al., 2016). To obtain the maximum economic benefits, camelina of a double‐ or relay‐cropping system, more consistent winter survival, and early flowering is required (Gesch et al., 2014). This is only possible if the genotype has strong winter survival potential in Northern Great Plain regions. The winter genotype Joelle has shown excellent winter survival and overwintering at extremely low temperature in field conditions (Gesch & Cermak, 2011; Gesch et al., 2018; Walia et al., 2018). Thus, our study was designed to elucidate the freezing tolerance mechanism that differentiates it from a highly freezing sensitive spring genotype C046. Identifying the loci regulating these differences will provide a valuable target for future breeding to enhance freezing tolerance in camelina and potentially other related brassica crops such as canola.

### 4.1 Efficacy of the phenotyping in our study

We repeatedly phenotyped all the F3 RIL families to select homozygous and segregating progeny for freezing tolerance. Additionally, we repeatedly phenotyped 254 of the original 255 F7 families derived by single seed decent from these F3 families. Thus, we are confident that the four F3 families chosen for the homozygosity mapping are homozygous for the freezing tolerance/susceptible trait. Previous studies suggested that freezing tolerance between the parents of these families was controlled by only one or two dominant genes (Horvath et al., 2019). This is clearly not the case. The discrepancy is most likely due the limited number of F2 progeny examined in the earlier study and the observation that there is considerable phenotypic plasticity in the response of lines that are segregating for freezing tolerance. The data here, with 9 lines out of 254 showing full parent‐like phenotypes consistently from the F3 through the F7 generation suggest that there may be as few as three co‐dominant genes that must be homozygous to give a parental phenotypic response to freezing stress.

### 4.2 Homozygosity mapping in other systems

Homozygosity mapping technology has previously been limited to human and animal studies. Several researchers used different approaches to find homozygous blocks and candidate genes with variable sized homozygous blocks/windows. In humans, homozygosity mapping identified regions and candidate genes for congenital heart disease (Gholipoorfeshkecheh et al., 2020), recessive genes for 13 different diseases (Hildebrandt et al. 2009), and candidate genes for the trait of hereditary hair loss (Zeinali et al., 2021). Although the size of each identified homozygous block varied significantly, in 93% of the cases the disease‐causing candidate genes were found within the identified homozygous blocks. Limited applications of homozygosity mapping have been reported in plants. Kumar et al. (2020) applied homozygosity mapping in pear plants to reveal their population history and trait architecture using SNP markers. In another study, Machado et al. (2016) used microsatellite markers to detect homozygosity and genetic variability in an F4 castor bean population without performing a genome‐wise scan. Li et al. (2021) used a modified homozygosity mapping approach to identify the gene underlying the barley variegation mutant luteostrians. However, to our knowledge, the application of homozygosity mapping to identify candidate genes for a non‐mutant trait from a bi‐parental segregating population has not been reported in any plants. We are the first attempting to use this relatively new technology to identify freezing tolerant genes in camelina.

### 4.3 High throughput sequencing ensures good quality markers for freezing tolerance or susceptibility

According to several studies, freezing tolerance in plants is a complex trait (Augustyniak et al., 2020; Huang et al., 2018; Wrucke et al., 2020). Phenotyping plants for complex traits such as freezing tolerance is difficult, highly labor intensive, and time consuming because one must screen multiple plants at a large scale (Huang et al., 2018). Thus, developing good quality molecular markers for freezing tolerance would be beneficial to breeders. A set of reliable molecular markers linked to freezing tolerance not only can reduce breeding time and space but also increase the efficacy of reliable selection (Huang et al., 2018). Our data identified multiple markers associated with several loci that appear to be linked to freezing tolerance in two camelina varieties. Additionally, we have identified over 126K markers that consistently differentiate the two parents used in our study. It is likely that these markers could also be used to identify other genes of interest controlling agronomically important traits that differ between these two varieties including flowering time, spring versus winter biotype, yield, seed size, and oil content/quality. However, the small size of the homozygous blocks in the F3 families that were fixed for freezing tolerance suggests that a fair number of the markers that differentiate the two parents may not be segregating as expected and should be further screened to identify those that show a probable Mendelian segregation pattern in a segregating population.

### 4.4 Homozygosity mapping reveals candidate region for detecting causal gene for freezing tolerance

In our study, we identified 617 markers that were homozygous in the parental varieties and that were already fixed for freezing tolerance in the F3 families. The majority of these markers mapped to two contigs that represent a large portion of chromosome 11 based on homology to the published reference genome. The contigs in question are large (18,617,245 bp for contig 2 and 7,057,017 bp for contig 25) and appear to be adjacent and contiguous with less than a 2000 base gap between them as based on the published reference sequence. However, not all markers within these contigs showed homozygosity in both parents and the F3 families. It is unlikely that the median density of homozygous markers (1155 bases between markers) was too sparse to clearly delineate homozygous blocks as crossover rates on average should be roughly 200 kb per every 1% crossovers (1 centimorgan) (Goodman et al., 1995). It is more likely that the error rate in calling homozygous markers was too high to accurately generate the likely existence of large homozygous blocks. That said, we did identify multiple stretches of closely linked homozygous markers on these two contigs that ranged in size from a few hundred bases to more than 20 kb in size. It is noteworthy that among the 22 genes with strong homology to sequences within or near these homozygous blocks, each of the two largest peaks contained a gene that was differentially expressed between the two parents following 8 weeks of cold acclimation or were differentially expressed in one or both parents as they cold acclimated over time (Anderson et al., 2022). Thus, this observation would be consistent with these homozygous regions having a higher‐than‐average role in freezing tolerance.

Genes that make up the *CBF* regulon are often key targets when studying freezing tolerance (Ding et al., 2019; Thomashow, 1990). Indeed, several association mapping studies for freezing tolerance in arabidopsis have identified a region that include the three *CBF* genes on chromosome 4 of arabidopsis (Gehan et al., 2015; Horton et al., 2016; Oakley et al., 2014). Intriguingly, two *CBF* genes are located on chromosome 11 in camelina. Although these genes were not within or adjacent to any long stretches of closely linked homozygous markers, the fact that these critical genes are co‐localized within the two contigs that host the majority of the homozygous markers means we cannot rule out the possibility that selection of strong freezing tolerance/susceptibility observed in the parental lines is not the result of genetic differences within these genes. However, although there is differential expression of the *CBF* genes between acclimated CO46 and Joelle, *CBF* is induced in both varieties by cold treatments (Anderson et al. 2022; Horvath et al., 2019).

We also examined other genes within or near homozygous blocks on chromosome 11 that were previously identified as having been associated with syntenic regions in other brassica species. Several quantitative trait loci (QTL) associated with freezing tolerance have been identified in arabidopsis on chromosomes 1, 4, and 5 (Horton et al. 2016). Intriguingly, the majority of the candidate genes identified in the arabidopsis study from both chromosome 4 and 5 mapped to syntenic regions on camelina chromosome 11 including *CBF*. However, the syntenic regions, although mapping relatively close to the homozygous blocks identified in our study, were not overlapping with them (within the 50 kb zones). Few QTL studies on freezing tolerance have been done in brassica crops where the QTL regions have been mapped to the reference genome. We found only one such study which identified a large region of Chromosome A08 of *Brassica napus* that was associated with differential freezing tolerance (Huang et al., 2018). Surprisingly, there are no large syntenic blocks between *B. napus* and camelina in chromosome A08. However, over 300 genes from chromosome 11 of camelina had their best matches to chromosome A08 of *B. napus* based on a BlastN analysis (data not shown), and there are genes within the large QTL on chromosome A08 that map to chromosome 11 of camelina. However, there were no obvious syntenic regions within the homozygous blocks on chromosome 11 that might suggest synteny of freezing tolerance genes between camelina and *B. napus*.

### 4.5 Possible candidate genes responsible for freezing tolerance/susceptibility

Our homozygosity mapping study detected 22 candidate genes associated with freezing tolerance mechanisms that were similar to sequences located within 5 kb upstream and downstream of homozygous blocks. Intriguingly, BlastN indicated multiple transcripts from chromosome 11 and 12 annotated as *cysteine‐rich RLK* had their top match to the second highest peak that was located on contig 25. Additionally, BlastN indicated three *receptor serine/threonine* kinase‐like protein transcripts that had their top match to sequences within this peak. Of the likely transcripts that mapped to this locus, one of the *receptor serine/threonine* kinase‐like transcripts was differentially expressed between the two parents. Also, several closely related *cysteine‐rich RLK* genes that mapped near this locus but not directly in it were also differentially expressed in previous studies (Anderson et al., 2022). Furthermore, this locus had the largest stretch of homozygous markers. Considering the above‐mentioned facts, we hypothesize that this locus may be one of the primary loci responsible for the differences in freezing tolerance between the two parental varieties.

The only gene located in our targeted locus on contig 25 that was clearly differentially expressed between the two parents, encodes a *receptor serine/threonine* kinase‐like protein and annotated as Csa11g022770. The most similar gene in arabidopsis is AT4G18250. According to the expression browser from The Arabidopsis Information Resource website (https://www.arabidopsis.org/), AT4G18250 is induced in the roots in response to salt stress and the leaves in response to light stress as well as following various biotic stress events. However, it was not shown as being induced by cold stress. Surprisingly, this gene, like the surrounding *cysteine‐rich RLK* genes, was induced in response to cold acclimating conditions in CO46, but not in Joelle. Interestingly, the arabidopsis orthologue of the *receptor serine/threonine* kinase genes were previously found to play a role in ABA signaling and plant abiotic stress response (Lim et al., 2015; Osakabe et al., 2010). The overexpression of *Serine/threonine kinase*‐based genes were reported in camelina (Anderson et al. 2022), in pepper (Tang et al., 2022), in arabidopsis (Mao et al., 2010), and in grapevine (Sawicki et al., 2019) during cold exposure/treatment. Intriguingly, modulating *serine/threonine kinase*‐based genes have shown to enhance freezing tolerance in arabidopsis (Ding et al., 2015; Mao et al., 2010) and in pepper (Lim et al., 2020). These strengthen our finding and hypothesis that this *serine/threonine kinase*‐based gene is probably involved in freezing tolerance and ABA signaling in camelina.

The other genes located within this peak encode *cysteine‐rich RLKs*. It should be noted though that the ∼50 kb homozygous region is too small to encode all of the genes that show high homology to this stretch of DNA. Indeed, an analysis of the sequence suggests it contains only one full‐length copy of a *cysteine‐rich RLK* gene that is highly similar to but not identical to the reference camelina transcript Csa11g022780 and several portions of other likely *cysteine‐rich RLK* sequences which could be multiply spliced. The fact that previously predicted *cysteine‐rich RLK* genes have their best matches to this region—many of which were preferentially expressed in the freezing sensitive CO46 parent, suggests that there may be some structural anomalies in the predicted location of these genes in the published camelina reference genome and possibly some expansion of the gene family resulting from mischaracterized splice variants of these genes. However, being a part of large *RLKs* family, *cysteine‐rich RLKs* were reported to have influence in growth and development in arabidopsis and can modulate abscisic acid (ABA) response and drought tolerance (Lu et al., 2016; Pelagio‐Flores et al., 2020; Zhang et al., 2013a, 2013b). ABA influences both drought and freezing tolerance through plant stomatal movement (Lu et al., 2016) and it has been reported that around 10% of genes that were expressed during drought induction were also induced during cold stress (Shinozaki et al., 2003). In addition, *cysteine‐rich* *RLK1* (*CRK1*) was shown to play a positive role in protecting plants from cold stress (Ye et al., 2017) and were also differentially expressed during cold treatment in tomato (Chen et al., 2015) and in *Medicago sativa* (Song et al., 2016). Expression of *cysteine‐rich RLK* proteins such as CRK4, CRK5, CRK19, and CRK20 were found to cause a hypersensitive response‐associated programmed cell death in arabidopsis (Burdiak et al., 2015; Lu et al., 2016; Wrzaczek et al., 2010). Thus, the presence of CRK gene may enhance freezing tolerance by increasing ABA production or may cause death of plants by negatively regulating the ABA pathway.

Even though the *cysteine‐rich RLKs* and *receptor serine/threonine kina*se genes from contig 25 seems to be the most reasonable candidate for freezing tolerance based on our finding and cited literature reviews, we cannot rule out other possible genes, particularly from the longest stretches of homozygous markers located in contig 2. Interestingly, majority of the seven homozygous blocks from contig 2 contained either a limited number of genes or genes with limited information or involvement with freezing tolerance trait (for instance, “Csa11g045260” and “Csa11g045270” genes appeared in one of our homozygous blocks but had no matched genes in arabidopsis). However, one block from contig 2 positioned between 9,648,098 and 9,698,140 contained a gene encoding a *rotamase cyclophilin 2* (Csa11g052140). The *rotamase cyclophilin 2* gene is analogous to arabidopsis AT3G56070 which contains a peptidyl‐prolyl *cis*–*trans* isomerase domain. This gene was previously reported to increase cold tolerance in arabidopsis and regulate plant growth and development (Weng et al., 2020). Another interesting gene from the largest homozygous peak of contig 2, is Csa11g052230, which is analogous to AT1G66100 and encodes a *plant thionin* function. Although we did not find any literature that shows it is involvement in cold/freezing tolerance, it is involved in defense responses and showed differential expression between the two parents. Taking these together, our results suggest two genes from contig 25 with *cysteine‐rich RLKs* and *receptor serine/threonine* kinase function and one gene from contig 2 with cyclophilin function may play a significant role in freezing tolerance in camelina. Further QTL and fine mapping study on the freezing tolerance trait in camelina may provide validation of the involvement of our identified genes in freezing tolerance mechanism in camelina.

Although there is high similarity between the gene sequences from the reference genome and the assemblies used for this study, there are significant differences as well. For example, multiple annotated genes mapping to two different chromosomes in the previously published reference sequence had their best matches to the limited number of *cysteine‐rich RLK* sequences and the *receptor serine/threonine kinase* gene located in the peak on contig 25. Such anomalies indicate the limitations of the existing available reference genome. Thus, our findings support the need for an improved camelina draft reference genome with maximum coverage that can deal with such situations efficiently. We expect to develop a new reference for the spring type CO46 and winter variety Joelle from our assemblies. The molecular markers developed for this study should prove invaluable for ordering the various contigs into chromosome‐level scaffolds.

## 5 CONCLUSION

Application of relatively new but effective homozygosity mapping technology can provide new insights for genetic studies in plants. Using homozygosity mapping derived markers highlighted regions along chromosome 11 of camelina that appear to differentiate the freezing tolerance differences between the spring biotype CO46 and the winter biotype Joelle. Candidate genes associated with these markers identified homozygous blocks containing multiple *cysteine‐rich RLK* genes and a *receptor serine/threonine* kinase gene that may be responsible for freezing tolerance differences in our F3 camelina population. Likewise, the differences in the *rotamase cyclophilin 2* gene could also be responsible for the variation in freezing tolerance between Joelle and CO46. However, the causative nature of these genes still needs to be confirmed by QTL and fine mapping studies and engineering lines camelina that over‐ or under‐express these genes. Additionally, we cannot rule out the possibility that other loci on portions of camelina chromosome 11 may also play a role in freezing tolerance/susceptibility. Thus, further studies are needed to test the hypothesis that these and other loci/genes might also be important in determining the relative freezing survival between these two divergent camelina varieties. Once we are certain about the roles these candidate genes play in freezing tolerance, it should be possible to manipulate those genes by transformation or CRISPR modification and increase freezing tolerance of other spring varieties of camelina. These candidate genes may also provide valuable resource to enhance freezing tolerance in other brassica species such as canola.

## AUTHOR CONTRIBUTIONS

**TM Shaikh**: Conceptualization; formal analysis; investigation; methodology; project administration; validation; visualization; writing–original draft; writing–review and editing. **Mukhlesur Rahman**: Conceptualization; formal analysis; funding acquisition; investigation; methodology; project administration; resources; supervision. **Timothy Smith**: Formal analysis; investigation; methodology; resources. **James V. Anderson**: Formal analysis; resources; writing–review and editing. **Wun S. Chao**: Conceptualization; formal analysis; investigation; resources; writing–review and editing. **David P. Horvath**: Conceptualization; data curation; formal analysis; funding acquisition; investigation; methodology; project administration; resources; software; supervision; validation; visualization; writing–original draft; writing–review and editing.

## CONFLICT OF INTEREST

The authors declare no conflict of interest.

## DISCLAIMER

Mention of trade names or commercial products in this publication is solely for the purpose of providing specific information and does not imply recommendation or endorsement by the U.S. Department of Agriculture. USDA is an equal opportunity provider and employer.

## Supporting information

Supplemental Material
Supplemental files can be downloaded from the following link: https://doi.org/10.5281/zenodo.7382879
Supplemental file S1: Fasta file of the reference sequence to which the short reads were mapped.
Supplemental file S2: Table of the number of raw and clean reads from each library as well as the percentage of reads that mapped to the reference sequence.
Supplemental file S3: Computer code used to clean the raw sequence data and to map it to the reference sequence.
Supplemental file S4: Table showing survival scores of selected lines for each of the 12 individuals in both experimental runs.
Supplemental file S5: VCF file generated from the mapping of the sequences from the 15 F3 families with the mapping quality for the 617 markers that were selected as being associated with the phenotype and their locations on the two contigs.

## References

[tpg220318-bib-0001] Abdullah, H. M. , Akbari, P. , Paulose, B. , Schnell, D. , Qi, W. , Park, Y. , Pareek, A. , & Dhankher, O. P. (2016). Transcriptome profiling of *Camelina sativa* to identify genes involved in triacylglycerol biosynthesis and accumulation in the developing seeds. Biotechnology for Biofuels and Bioproducts, 9, 136. 10.1186/s13068-016-0555-5 PMC4932711 27382413

[tpg220318-bib-0002] Anderson, J. V. , Horvath, D. P. , Doğramaci, M. , Dorn, K. M. , Chao, W. S. , Watkin, E. E. , Hernandez, A. G. , Marks, M. D. , & Gesch, R. (2018). Expression of FLOWERING LOCUS C and a frame shift mutation of this gene on chromosome 20 differentiate a summer‐ and winter‐annual biotype of *Camelina sativa* . Plant Direct, 2, e00060. 10.1002/pld3.60 31245730 PMC6508819

[tpg220318-bib-0003] Anderson, J. V. , Neubauer, M. , Horvath, D. P. , Chao, W. S. , & Berti, M. T. (2022). Analysis of *Camelina sativa* transcriptomes identified specific transcription factors and processes associated with freezing tolerance in a winter biotype. Industrial Crops and Products, 177, 114414. 10.1016/j.indcrop.2021.114414

[tpg220318-bib-0004] Anderson, J. V. , Wittenberg, A. , Li, H. , & Berti, M. T. (2019). High throughput phenotyping of Camelina sativa seeds for crude protein, total oil, and fatty acids profile by near infrared spectroscopy. Industrial Crops and Products, 137, 501–507. 10.1016/j.Indcrop.2019.04.075

[tpg220318-bib-0005] Augustyniak, A. , Pawłowicz, I. , Lechowicz, K. , Izbiańska‐Jankowska, K. , Arasimowicz‐Jelonek, M. , Rapacz, M. , Perlikowski, D. , & Kosmala, A. (2020). Freezing tolerance of *Lolium multiflorum*/*Festuca arundinacea* introgression forms is associated with the high activity of antioxidant system and adjustment of photosynthetic activity under cold acclimation. International Journal of Molecular Science, 21, 5899. 10.3390/ijms21165899 PMC7460622 32824486

[tpg220318-bib-0006] Berti, M. , Gesch, R. , Eynck, C. , Anderson, J. , & Cermak, S. (2016). Camelina uses, genetics, genomics, production, and management. Industrial Crops and Products, 94, 690–710. 10.1016/j.indcrop.2016.09.034

[tpg220318-bib-0007] Berti, M. T. , Samarappuli, D. , Johnson, B. , & Gesch, R. W. (2017a). Integrating winter camelina into maize and soybean cropping systems. Industrial Crops and Products, 107, 595–601. 10.1016/j.indcrop.2017.06.014

[tpg220318-bib-0008] Bouby, L. (1998). Two early finds of gold‐of‐pleasure *(Camelina* sp.) in middle Neolithic and Chalcolithic sites in western France. Antiquity, 72(276), 391–398. 10.1017/S0003598X0008666X

[tpg220318-bib-0009] Burdiak, P. , Rusaczonek, A. , Witoń, D. , Głów, D. , & Karpiński, S. (2015). Cysteine‐rich receptor‐like kinase CRK5 as a regulator of growth, development, and ultraviolet radiation responses in *Arabidopsis thaliana* . Journal of Experimental Botany, 66(11), 3325–3337. 10.1093/jxb/erv143 25969551 PMC4449547

[tpg220318-bib-0010] Bushnell, B. (2014). BBMap: A fast, accurate, splice‐aware aligner. Lawrence Berkeley National Lab (LBNL).

[tpg220318-bib-0011] Chao, W. S. , Wang, H. , Horvath, D. P. , & Anderson, J. V. (2019). Selection of endogenous reference genes for qRT‐PCR analysis in *Camelina sativa* and identification of FLOWERING LOCUS C allele‐specific markers to differentiate summer‐ and winter‐biotypes. Industrial Crops and Products, 129, 495–502. 10.1016/j.indcrop.2018.12.017

[tpg220318-bib-0012] Chaudhary, R. , Koh, C. S. , Kagale, S. , Tang, L. , Wu, S. W. , Lv, Z. , Mason, A. S. , Sharpe, A. G. , Diederichsen, A. , & Parkin, I. A. P. (2020). Assessing diversity in the *Camelina* genus provides insights into the genome structure of *Camelina sativa* . Genes Genomics Genetics, 10(4), 1297–1308. 10.1534/g3.119.400957 PMC7144077 32046969

[tpg220318-bib-0013] Chen, H. , Chen, X. , Chen, D. , Li, J. , Zhang, Y. , & Wang, A. (2015). A comparison of the low temperature transcriptomes of two tomato genotypes that differ in freezing tolerance: *Solanum lycopersicum* and *Solanum habrochaites* . BMC Plant Biology, 15, 132. 10.1186/s12870-015-0521-6 26048292 PMC4458020

[tpg220318-bib-0014] Chen, K. , Du, L. , & Chen, Z. (2003). Sensitization of defense responses and activation of programmed cell death by a pathogen‐induced receptor‐like protein kinase in *Arabidopsis* . Plant Molecular Biology, 53, 61–74. 10.1023/B:PLAN.0000009265.72567.58 14756307

[tpg220318-bib-0015] Ding, Y. , Li, H. , Zhang, X. , Xie, Q. , Gong, Z. , & Yang, S. (2015). OST1 kinase modulates freezing tolerance by enhancing ICE1 stability in *Arabidopsis* . Developmental Cell, 32, 278–289. 10.1016/j.devcel.2014.12.023 25669882

[tpg220318-bib-0016] Ding, Y. , Shi, Y. , & Yang, S. (2019). Advances and challenges in uncovering cold tolerance regulatory mechanisms in plant. New Phytologist, 222(4), 1690–1704. 10.1111/nph.15696 30664232

[tpg220318-bib-0017] Downey, R. K. , & Rakow, G. F. W. (1987). Rapeseed and mustard. In W. R. Fehr (Ed.), Principles of cultivar development. Volume 2–Crop species (pp. 437–486). Macmillan Publishing Company.

[tpg220318-bib-0018] Doyle, J. J. , & Doyle, J. L. (1990). Isolation of plant DNA from fresh tissue. Focus, 12(1), 13–15.

[tpg220318-bib-0019] Eberle, C. A. , Thom, M. D. , Nemec, K. T. , Forcella, F. , Lundgren, J. G. , Gesch, R. W. , Riedell, W. E. , Papiernik, S. K. , Wagner, A. , Peterson, D. H. , & Eklund, J. J. (2015). Using pennycress, camelina, and canola cash cover crops to provision pollinators. Industrial Crops and Products, 75, 20–25. 10.1016/j.indcrop.2015.06.026

[tpg220318-bib-0020] Fiebelkorn, D. , Horvath, D. , & Rahman, M. (2018). Genome‐wide association study for electrolyte leakage in rapeseed/canola (*Brassica napus* L.). Molecular Breeding, 38, 129. 10.1007/s11032-018-0892-0

[tpg220318-bib-0021] Gallant, A. L. , Euliss, N. H. , & Browning, Z. (2014). Mapping large‐area landscape suitability for honey bees to assess the influence of land‐use change on sustainability of national pollination services. PLoS ONE, 9(6), e99268. 10.1371/journal.pone.0099268 24919181 PMC4053381

[tpg220318-bib-0022] Gehan, M. A. , Park, S. , Gilmour, S. J. , An, C. , Lee, C.‐M. , & Thomashow, M. F. (2015). Natural variation in the C‐repeat binding factor cold response pathway correlates with local adaptation of Arabidopsis ecotypes. The Plant Journal, 84(4), 682–693. 10.1111/tpj.13027 26369909

[tpg220318-bib-0023] Gesch, R. (2014). Influence of genotype and sowing date on camelina growth and yield in the north central U.S. Industrial Crops and Products, 54, 209–215. 10.1016/j.indcrop.2014.01.034

[tpg220318-bib-0024] Gesch, R. W. , & Archer, D. W. (2013). Double‐cropping with winter camelina in the northern corn belt to produce fuel and food. Industrial Crops and Products, 44, 718–725. 10.1016/j.indcrop.2012.05.023

[tpg220318-bib-0025] Gesch, R. W. , Archer, D. W. , & Berti, M. T. (2014). Dual cropping winter camelina with soybean in the northern corn belt. Agronomy Journal, 106(5), 1735–1745. 10.2134/agronj14.0215

[tpg220318-bib-0026] Gesch, R. W. , & Cermak, S. C. (2011). Sowing date and tillage effects on fall‐seeded camelina in the northern corn belt. Agronomy Journal, 103(4), 980–987. 10.2134/agronj2010.0485

[tpg220318-bib-0027] Gesch, R. W. , Matthees, H. L. , Alvarez, A. L. , & Gardner, R. D. (2018). Winter camelina: Crop growth, seed yield, and quality response to cultivars and seeding rate. Agronomy Journal, 58, 2089–2098. 10.2135/cropsci2018.01.0018

[tpg220318-bib-0028] Gholipoorfeshkecheh, R. , Agarwala, S. , Krishnappa, S. , Savitha, M. R. , & Ramachandra, N. B. (2020). Whole‐exome sequencing and homozygosity mapping identify variants in NCOR1 and MAP2K3 associated with nonsyndromic congenital heart defects. Egyptian Journal of Medical Human Genetics, 21, 58. 10.1186/s43042-020-00101-4

[tpg220318-bib-0029] Goodman, H. M. , Ecker, J. R. , & Dean, C. (1995). The genome of *Arabidopsis thaliana* . Proceedings of the National Academy of Sciences, 92(24), 10831–10835. 10.1073/pnas.92.24.10831 PMC40525 7479893

[tpg220318-bib-0030] Guy, C. L. , Anderson, J. V. , Haskell, D. W. , & Li, Q.‐B. (1994). CAPs, cors, dehydrins, and molecular chaperones: Their relationship with low temperature responses in spinach. In J. H. Cherry (Ed.), Biochemical and cellular mechanisms of stress tolerance in plants, NATO‐ASI series, (Vol. H86, pp. 479–499). Springer‐Verlag.

[tpg220318-bib-0084] Horton, M. W. , Willems, G. , Sasaki, E. , Koornneef, M. , & Nordborg, M. (2016). The genetic architecture of freezing tolerance varies across the range of Arabidopsis thaliana. Plant, Cell & Environment, 39, 2570–2579. 10.1111/pce.12812 27487257

[tpg220318-bib-0031] Horvath, D. , Anderson, J. V. , Chao, W. S. , Zheng, P. , Buchwaldt, M. , Parkin, I. A. P. , & Dorn, K. (2019). Genes associated with chloroplasts and hormone‐signaling, and transcription factors other than CBFs are associated with differential survival after low temperature treatments of Camelina sativa biotype. PLoS ONE, 14(5), e0217692. 10.1371/journal.pone.0217692 31150478 PMC6544293

[tpg220318-bib-0032] Huang, Z. , Zhao, N. , Qin, M. , & Xu, A. (2018). Mapping of quantitative trait loci related to cold tolerance in *Brassica napus* L. Journal of Plant Physiology, 231, 147–154. 10.1016/j.jplph.2018.09.012 30268695

[tpg220318-bib-0033] Jaglo‐Ottosen, K. R. , Gilmour, S. J. , Zarka, D. G. , Schabenberger, O. , & Thomashow, M. F. (1998). Arabidopsis CBF1 overexpression induces COR genes and enhances freezing tolerance. Science, 280, 104–106. 10.1126/science.280.5360.104 9525853

[tpg220318-bib-0034] Kagale, S. , Koh, C. , Nixon, J. , Bollina, V. , Clarke, W. E. , Tuteja, R. , Spillane, C. , Robinson, S. J. , Links, M. G. , Clarke, C. , Higgins, E. E. , Huebert, T. , Sharpe, A. G. , & Parkin, I. A. P. (2014). The emerging biofuel crop Camelina sativa retains a highly undifferentiated hexaploid genome structure. Nature Communications, 5, 3706. 10.1038/ncomms4706 PMC4015329 24759634

[tpg220318-bib-0035] Kagale, S. , Nixon, J. , Khedikar, Y. , Pasha, A. , Provart, N. J. , Clarke, W. E. , Bollina, V. , Robinson, S. J. , Coutu, C. , Hegedus, D. D. , Sharpe, A. G. , & Parkin, I. A. P. (2016). The developmental transcriptome atlas of the biofuel crop *Camelina sativa* . Plant Journal, 88, 879–894. 10.1111/tpj.13302 27513981

[tpg220318-bib-0036] Kumar, S. , Deng, C. H. , Hunt, M. , Kirk, C. , Wiedow, C. , Rowan, D. , Wu, J. , & Brewer, L. (2020). Homozygosity mapping reveals population history and trait architecture in self‐incompatible pear (Pyrus spp.). Frontiers Plant Science, 11, 590846. 10.3389/fpls.2020.590846 PMC7813798 33469460

[tpg220318-bib-0037] Li, H. , Hu, X. , Lovell, J. T. , Grabowski, P. P. , Mamidi, S. , Chen, C. , Amirebrahimi, M. , Kahanda, I. , Mumey, B. , Barry, K. , Kudrna, D. , Schmutz, J. , Lachowiec, J. , & Lu, C. (2021). Genetic dissection of natural variation in oilseed traits of camelina by whole‐genome resequencing and QTL mapping. Plant Genome, 14(2), e20110. 10.1002/tpg2.20110 34106529 PMC12806864

[tpg220318-bib-0038] Li, M. , Guo, G. , Pidon, H. , Melzer, M. , Prina, A. R. , Börner, T. , & Stein, N. (2021). ATP‐dependent Clp protease subunit C1, HvClpC1, is a strong candidate gene for barley variegation mutant luteostrians as revealed by genetic mapping and genomic re‐sequencing. Frontiers in Plant Science, 12, 664085. 10.3389/fpls.2021.664085 33936155 PMC8086601

[tpg220318-bib-0039] Li, Z. , & Xu, Y. (2021). Bulk segregation analysis in the NGS era: A review of its teenage years. The Plant Journal, 109(6), 1355–1374. 10.1111/tpj.15646 34931728

[tpg220318-bib-0040] Lim, C. W. , Baek, W. , Jung, J. , Kim, J. H. , & Lee, S. C. (2015). Function of ABA in stomatal defense against biotic and drought stresses. International Journal of Molecular Sciences, 16(7), 15251–15270. 10.3390/ijms160715251 26154766 PMC4519898

[tpg220318-bib-0041] Lim, J. , Lim, C. W. , & Lee, S. C. (2020). Pepper novel serine‐threonine kinase CaDIK1 regulates drought tolerance via modulating ABA sensitivity. Frontier Plant Science, 11, 1133. 10.3389/fpls.2020.01133 PMC7390950 32793275

[tpg220318-bib-0042] Liu, X. , Brost, J. , Hutcheon, C. , Guilfoil, R. , & Wilson, A. K. (2012). Transformation of the oilseed crop *Camelina sativa* by *Agrobacterium*‐mediated floral dip and simple large‐scale screening of transformants. In Vitro Cellular & Developmental Biology–Plant, 48, 462–468. 10.1007/s11627-012-9459-7

[tpg220318-bib-0043] Lu, C. , & Kang, J. (2008). Generation of transgenic plants of a potential oilseed crop *Camelina sativa* by *Agrobacterium*‐mediated transformation. Plant Cell Reports, 27, 273–278. 10.1007/s00299-007-0454-0 17899095

[tpg220318-bib-0044] Lu, K. , Liang, S. , Wu, Z. , Bi, C. , Yu, Y.‐T. , Wang, X.‐F. , & Zhang, D.‐P. (2016). Overexpression of an Arabidopsis cysteine‐rich receptor‐like protein kinase, CRK5, enhances abscisic acid sensitivity and confers drought tolerance. Journal of Experimental Botany, 67(17), 5009–5027. 10.1093/jxb/erw266 27406784 PMC5014153

[tpg220318-bib-0045] Luo, Z. , Szczepanek, A. , & Abdel‐Haleem, H. (2020). Genome‐wide association study (GWAS) analysis of camelina seedling germination under salt stress condition. Agronomy, 10(9), 1444. 10.3390/agronomy10091444

[tpg220318-bib-0046] Machado, E. L. , Silva, S. A. , Fernandes, L. D. S. , & Brasileiro, H. S. (2016). Genetic variability and homozygosity in a F4 castor bean population by microsatellite markers. Bragantia, 75, 307–313. 10.1590/1678-4499.536

[tpg220318-bib-0047] Mahmoud, M. , Zywicki, M. , Twardowski, T. , & Karlowski, W. M. (2019). Efficiency of PacBio long read correction by 2nd generation Illumina sequencing. Genomics, 111, 43–49. 10.1016/j.ygeno.2017.12.011 29268960

[tpg220318-bib-0048] Malik, M. R. , Tang, J. , Sharma, N. , Burkitt, C. , Ji, Y. , Mykytyshyn, M. , Bohmert‐Tatarev, K. , Peoples, O. , & Snell, K. D. (2018). *Camelina sativa*, an oilseed at the nexus between model system and commercial crop. Plant Cell Reports, 37(10), 1367–1381. 10.1007/s00299-018-2308-3 29881973

[tpg220318-bib-0049] Mao, X. , Zhang, H. , Tian, S. , Chang, X. , & Jing, R. (2010). TaSnRK2.4, an SNF1‐type serine/threonine protein kinase of wheat (*Triticum aestivum* L.), confers enhanced multistress tolerance in *Arabidopsis* . Journal of Experimental Botany, 61(3), 683–696. 10.1093/jxb/erp331 20022921 PMC2814103

[tpg220318-bib-0050] Marçais, G. , Delcher, A. L. , Phillippy, A. M. , Coston, R. , Salzberg, S. L. , & Zimin, A. (2018). MUMmer4: A fast and versatile genome alignment system. PLoS Computational Biology, 14(1), e1005944. 10.1371/journal.pcbi.1005944 29373581 PMC5802927

[tpg220318-bib-0051] McVay, K. A. , & Lamb, P. F. (2008). Camelina production in Montana. Montana State University. http://store.msuextension.org/publications/AgandNaturalResources/MT200701AG.pdf

[tpg220318-bib-0052] Michelmore, R. W. , Paran, I. , & Kesseli, R. V. (1991). Identification of markers linked to disease resistance genes by bulked segregant analysis: A rapid method to detect markers in specific genomic regions by using segregating populations. Proceedings National Academy of Sciences USA, 88, 9828–9832. 10.1073/pnas.88.21.9828 PMC52814 1682921

[tpg220318-bib-0053] Moore, R. H. , Thornhill, K. L. , Weinzierl, B. , Sauer, D. , D'ascoli, E. , Kim, J. , Lichtenstern, M. , Scheibe, M. , Beaton, B. , Beyersdorf, A. J. , Barrick, J. , Bulzan, D. , Corr, C. A. , Crosbie, E. , Jurkat, T. , Martin, R. , Riddick, D. , Shook, M. , Slover, G. , … Anderson, B. E. (2017). Biofuel blending reduces particle emissions from aircraft engines at cruise conditions. Nature, 543, 411–415. 10.1038/nature21420 28300096 PMC8025803

[tpg220318-bib-0054] Oakley, C. G. , Ågren, J. , Atchison, R. A. , & Schemske, D. W. (2014). QTL mapping of freezing tolerance: Links to fitness and adaptive trade‐offs. Molecular Ecology, 23(17), 4304–4315. 10.1111/mec.12862 25039860

[tpg220318-bib-0055] Osakabe, Y. , Mizuno, S. , Tanaka, H. , Maruyama, K. , Osakabe, K. , Todaka, D. , Fujita, Y. , Kobayashi, M. , Shinozaki, K. , & Yamaguchi‐Shinozaki, K. (2010). Overproduction of the membrane‐bound receptor‐like protein kinase 1, RPK1, enhances abiotic stress tolerance in *Arabidopsis* . Journal of Biological Chemistry, 285(12), 9190–9201. 10.1074/jbc.M109.051938 20089852 PMC2838338

[tpg220318-bib-0056] Pearce, R. (2001). Plant freezing and damage. Annals of Botany, 87(4), 417–424. 10.1006/anbo.2000.1352

[tpg220318-bib-0057] Pelagio‐Flores, R. , Muñoz‐Parra, E. , Barrera‐Ortiz, S. , Ortiz‐Castro, R. , Saenz‐Mata, J. , Ortega‐Amaro, M. A. , Jiménez‐Bremont, J. F. , & López‐Bucio, J. (2020). The cysteine‐rich receptor‐like protein kinase CRK28 modulates *Arabidopsis* growth and development and influences abscisic acid response. Planta, 251(1), 2. 10.1007/s00425-019-03296-y 31776759

[tpg220318-bib-0058] Rasheed, A. , Hao, Y. , Xia, X. , Khan, A. , Xu, Y. , Varshney, R. K. , & He, Z. (2017). Crop breeding chips and genotyping platforms: Progress, challenges, and perspectives. Molecular Plant, 10(8), 1047–1064. 10.1016/j.molp.2017.06.008 28669791

[tpg220318-bib-0059] Rhoads, A. , & Au, K. F. (2015). PacBio sequencing and its applications. Genomics, Proteomics & Bioinformatics, 13(5), 278–289. 10.1016/j.gpb.2015.08.002 PMC4678779 26542840

[tpg220318-bib-0060] Saucke, H. , & Ackermann, K. (2006). Weed suppression in mixed cropped grain peas and false flax (*Camelina sativa*). Weed Research, 46(6), 453–461. 10.1111/j.1365-3180.2006.00530.x

[tpg220318-bib-0061] Sawicki, M. , Rondeau, M. , Courteaux, B. , Rabenoelina, F. , Guerriero, G. , Gomès, E. , Soubigou‐Taconnat, L. , Balzergue, S. , Clément, C. , Barka, E. A. , Vaillant‐Gaveau, N. , & Jacquard, C. (2019). On a cold night: Transcriptomics of grapevine flower unveils signal transduction and impacted metabolism. International Journal of Molecular Sciences, 20, 1130. 10.3390/ijms20051130 30841651 PMC6429367

[tpg220318-bib-0063] Seelow, D. , & Schuelke, M. (2012). Homozygosity Mapper 2012—Bridging the gap between homozygosity mapping and deep sequencing. Nucleic Acids Research, 40, 516–520. 10.1093/nar/gks487 PMC3394249 22669902

[tpg220318-bib-0064] Shinozaki, K. , Yamaguchi‐Shinozaki, K. , & Seki, M. (2003). Regulatory network of gene expression in the drought and cold stress responses. Current Opinion in Plant Biology, 6, 410–417. 10.1016/S1369-5266(03)00092-X 12972040

[tpg220318-bib-0065] Shonnard, D. R. , Williams, L. , & Kalnes, T. N. (2010). Camelina‐derived jet fuel and diesel: Sustainable advanced biofuels. Environmental Progress & Sustainable Energy, 3, 382–392. 10.1002/ep.10461

[tpg220318-bib-0066] Song, L. , Jiang, L. , Chen, Y. , Shu, Y. , Bai, Y. , & Guo, C. (2016). Deep‐sequencing transcriptome analysis of field‐grown *Medicago sativa* L. crown buds acclimated to freezing stress. Functional & Integrative Genomics, 16(5), 495–511. 10.1007/s10142-016-0500-5 27272950

[tpg220318-bib-0067] Soorni, J. , Kazemitabar, S. , Kahrizi, D. , Dehestani, A. , & Bagheri, N. (2017). Screening of camelina (*Camelina sativa* L.) double haploid lines for freezing tolerance in the seedling stage. Genetica, 49, 173–181. 10.2298/GENSR1701173S

[tpg220318-bib-0068] Sutka, J. (2001). Genes for frost tolerance in wheat. Euphytica, 119, 169–177. 10.1023/A:1017520720183

[tpg220318-bib-0069] Tang, B. , Xie, L. , Yang, H. , Li, X. , Chen, Y. , Zou, X. , Liu, F. , & Dai, X. (2022). Analysis of the expression and function of key genes in pepper under low‐temperature stress. Frontier Plant Science, 13, 852511. 10.3389/fpls.2022.852511 PMC9116226 35599873

[tpg220318-bib-0070] Thomashow, M. F. (1990). Molecular genetics of cold acclimation in higher plants. Advances in Genetics, 28, 99–131. 10.1016/S0065-2660(08)60525-8

[tpg220318-bib-0071] Thomashow, M. F. (1999). Plant cold acclimation: Freezing tolerance genes and regulatory mechanisms. Annual Review Plant Physiology & Plant Molecular Biology, 50, 571–599. 10.1146/annurev.arplant.50.1.571 15012220

[tpg220318-bib-0072] Vollmann, J. , Moritz, T. , Kargl, C. , Baumgartner, S. , & Wagentristl, H. (2007). Agronomic evaluation of camelina genotypes selected for seed quality characteristics. Industrial Crops and Products, 26, 270–277. 10.1016/j.indcrop.2007.03.017

[tpg220318-bib-0073] Walia, M. K. , Wells, M. S. , Cubins, J. , Wyse, D. , Gardner, R. D. , Forcella, F. , & Gesch, R. (2018). Winter camelina seed yield and quality responses to harvest time. Industrial Crops and Products, 124, 765–775. 10.1016/j.indcrop.2018.08.025

[tpg220318-bib-0074] Wang, Z. , Yu, A. , Li, F. , Xu, W. , Han, B. , Cheng, X. , & Liu, A. (2021). Bulked segregant analysis reveals candidate genes responsible for dwarf formation in woody oilseed crop castor bean. Scientific Reports, 11, 6277. 10.1038/s41598-021-85644-1 33737619 PMC7973431

[tpg220318-bib-0075] Weng, Y. , Ge, L. , Jia, S. , Mao, P. , & Ma, X. (2020). Cyclophilin AtROC1S58F confers Arabidopsis cold tolerance by modulating jasmonic acid signaling and antioxidant metabolism. Plant Physiology and Biochemistry, 152, 81–89. 10.1016/j.plaphy.2020.04.012 32388423

[tpg220318-bib-0076] Wilke, B. J. , & Snapp, S. S. (2008). Winter cover crops for local ecosystems: Linking plant traits and ecosystem function. Journal of the Science of Food and Agriculture, 2008, 88, 551–557. 10.1002/jsfa.3149

[tpg220318-bib-0077] Wrucke, D. F. , Talukder, Z. I. , & Rahman, M. (2020). Genome‐wide association study for frost tolerance in rapeseed/canola (*Brassica napus*) under simulating freezing conditions. Plant Breeding, 139(2), 356–367. 10.1111/pbr.12771

[tpg220318-bib-0078] Wrzaczek, M. , Brosché, M. , Salojärvi, J. , Kangasjärvi, S. , Idänheimo, N. , Mersmann, S. , Robatzek, S. , Karpiński, S. , Karpińska, B. , & Kangasjärvi, J. (2010). Transcriptional regulation of the CRK/DUF26 group of receptor‐like protein kinases by ozone and plant hormones in *Arabidopsis* . BMC Plant Biology, 10, 95. 10.1186/1471-2229-10-95 20500828 PMC3095361

[tpg220318-bib-0079] Ye, Y. , Ding, Y. , Jiang, Q. , Wang, F. , Sun, J. , & Zhu, C. (2017). The role of receptor‐like protein kinases (RLKs) in abiotic stress response in plants. Plant Cell Reports, 36(2), 235–242. 10.1007/s00299-016-2084-x 27933379

[tpg220318-bib-0080] Zeinali, S. , Youssefian, L. , Vahidnezhad, H. , Saeidian, A. H. , Sotoudeh, S. , Bagherian, H. , & Uitto, J. (2021). Autozygosity mapping by genome‐wide single nucleotide polymorphism array identifies a novel homozygous HR mutation in a consanguineous family with universal hereditary hair loss. International Journal of Dermatology and Venereology, 4(2), 82–85. 10.1097/JD9.0000000000000168

[tpg220318-bib-0081] Zhang, X. , Han, X. , Shi, R. , Yang, G. , Qi, L. , Wang, R. , & Li, G. (2013a). Arabidopsis cysteine‐rich receptor‐like kinase 45 positively regulates disease tolerance to *Pseudomonas syringae* . Plant Physiology and Biochemistry, 73, 383–391. 10.1016/j.plaphy.2013.10.024 24215930

[tpg220318-bib-0082] Zhang, X. , Yang, G. , Shi, R. , Han, X. , Qi, L. , Wang, R. , Xiong, L. , & Li, G. (2013b). Arabidopsis cysteine‐rich receptor‐like kinase 45 functions in the responses to abscisic acid and abiotic stresses. Plant Physiology and Biochemistry, 67, 189–198. 10.1016/j.plaphy.2013.03.013 23583936

