# Supplementary material for: Homozygosity mapping identified loci and candidate genes responsible for freezing tolerance in Camelina sativa
Source: Plant Genome. 2023 Mar 9;16(2):e20318. doi: 10.1002/tpg2.20318 (PMC12807280; doi:10.1002/tpg2.20318)
Supplement: Supplementary file 1 [file TPG2-16-e20318-s001.docx]

Supplemental Material

Supplemental files can be downloaded from the following link:

https://doi.org/10.5281/zenodo.7382879

Supplemental file S1: Fasta file of the reference sequence to which the short reads were mapped.

Supplemental file S2: Table of the number of raw and clean reads from each library as well as the percentage of reads that mapped to the reference sequence.

Supplemental file S3: Computer code used to clean the raw sequence data and to map it to the reference sequence.

Supplemental file S4: Table showing survival scores of selected lines for each of the 12 individuals in both experimental runs.

Supplemental file S5: VCF file generated from the mapping of the sequences from the 15 F3 families with the mapping quality for the 617 markers that were selected as being associated with the phenotype and their locations on the two contigs.
